# Autophagy inhibition in pancreatic cancer: hope or hype?

**DOI:** 10.1080/27694127.2026.2721748

**Published:** 2026-08-27

**Authors:** Emma Guilbaud, Edna Cukierman, Igor Astsaturov, Lorenzo Galluzzi

**Affiliations:** a Cancer Signaling and Microenvironment Program, Fox Chase Cancer Center, Philadelphia, PA, USA; b Marvin and Concetta Greenberg Pancreatic Cancer Institute, Fox Chase Cancer Center, Philadelphia, PA, USA

**Keywords:** Hydroxychloroquine, immune checkpoint inhibitors, MEK, MHC class I, trametinib, type I interferon

## Abstract

Autophagy promotes tumor progression and immune evasion by numerous mechanisms, representing a promising target for novel (immuno)therapeutic interventions in multiple oncological indications. An expanding literature, however, suggests that safely and effectively targeting autophagy in the clinic remains challenging, especially in populations as complex to manage as patients with pancreatic cancer.

While proficient autophagic responses in healthy cells most often antagonize malignant transformation, largely reflecting the ability of autophagy to sustain physiological homeostasis, transformed cells generally harness autophagic responses in support of tumor progression, resistance to therapy and immune evasion [1,2]. Thus, over the past two decades, autophagy has stood out as a promising target for the development of novel cancer (immuno)therapeutics [1]. Such a prospect was especially compelling for hard-to-treat tumors such as PDAC (pancreatic ductal adenocarcinoma) [3]. Indeed, PDAC is an aggressive epithelial malignancy characterized by frequent alterations in *KRAS* (KRas proto-oncogene, GTPase), early local and systemic dissemination, profound stromal desmoplasia, intratumoral vascular collapse, and extensive metabolic plasticity, a panel of features associated with limited sensitivity to current therapeutic options (which are dictated by resectability at presentation but generally incorporate aggressive chemotherapy) and an extremely poor prognosis (~13% 5-year survival rate) [3].

Proficient autophagic responses in PDAC cells have consistently been shown to underlie tumor progression *in vivo* [4–6], limit the efficacy of conventional chemotherapeutics including gemcitabine [7], and support dormancy upon loss of mutant KRAS expression [5]. Moreover, PDAC progression in mice appears to rely (at least in part) on metabolic circuits involving autophagy activation in pancreatic CAFs (cancer-associated fibroblasts), which support PDAC cells by secreting alanine and nucleosides into the TME (tumor microenvironment) [8,9], releasing cytokines that rewire nucleotide metabolism in malignant cells [10], as well as by proline-dependent collagen deposition [11]. Finally, autophagy has been shown to effectively degrade MHC class I molecules [12], promote the exposure of anti-phagocytic signals [13], limit the cytosolic availability of nuclear and mitochondrial nucleic acids promoting interferon responses [14,15], and counteract the cytotoxic effects of immune effector molecules including GZMB (granzyme B) [16], globally endowing PDAC cells with pronounced immunoevasive features. Accordingly, multiple preclinical studies in mouse models of PDAC have reported considerable therapeutic benefits from concomitantly inhibiting the signal transduction cascades elicited by mutant KRAS and autophagy [17–20].

Based on these preclinical findings, multiple clinical trials have begun investigating the safety and therapeutic efficacy of clinical grade autophagy blockers – most often lysosomal inhibitors like CQ (chloroquine), HCQ (hydroxychloroquine) and derivatives thereof – in patients with various malignancies including PDAC. Despite sporadic signs of activity mostly limited to the pre-operative setting or fairly transitory (and not translating into – or not poised to establish – a *bona fide* survival advantage) [21–26], however, these clinical trials globally failed to demonstrate a therapeutic benefit for autophagy inhibitors (used alone or combined with other therapeutic agents) in patients with PDAC (Table 1). In this setting, clinical findings ranged indeed from negligible (if not fully absent) activity [24,27–31] to overt toxicity issues calling for treatment discontinuation and (at least in one case) premature trial termination [24,32–34].

**Table 1.** Clinical trials testing HCQ in patients with PDAC.

Indication	Phase	Nº	Co-treatment	Effect	Notes	NCT	Ref.
Advanced/metastatic	I	9	Gemcitabine	Reported median PFS of 4.0 months and median OS of 7.6 months	Single-arm study, documented no dose-limiting toxicities as defined in the study protocol	NCT01777477	[22]
Advanced/metastatic	II	10	Gemcitabine Nab-paclitaxel Paricalcitol	Reported median PFS of 6.0 months and median OS of 6.8 months	Single-arm study, documented serious adverse events in 30% of patients	NCT04524702	N/A
Advanced/metastatic	II	40	mFOLFIRINOX	Reported median PFS of 7.4 months and median OS of 14.8 months	Single-arm study, documented dose-limiting toxicities in 10% of patients in the HCQ arm	NCT05083780	[25]
Advanced/metastatic	II	112	Gemcitabine nab-Paclitaxel	Reported median PFS of 5.7 *vs*. 6.4 months and median OS of 11.1 *vs*. 12.1 months in the HCQ *vs*. non-HCQ arm	Two-arm study, documented increased rates of serious adverse events in the HCQ *vs*. non-HCQ arm	NCT01506973	[24]
Advanced/metastatic	N/A	34	Trametinib Palbociclib [1]	Reported median PFS of 52 *vs*. 56 days and median OS of 68 *vs*. 195 days in the HCQ *vs*. palbociclib arm	Retrospective case series comparing HCQ to palbociclib as partner for trametinib	N/A	[28]
Previously untreated, non-metastatic	II	12	Cisplatin Gemcitabine Nab-paclitaxel	Reported CA19-9 normalization in 36% of patients, OS data has not been disseminated [2]	Single-arm study, documented serious adverse events in >30% of patients	NCT04669197	[26]
Previously treated, metastatic	I	14	Cobimetinib	Reported median PFS of 7.7 weeks and median OS of 20.7 weeks	Single-arm study, prematurely terminated owing to toxicity concerns	NCT04214418	[32]
Previously treated, metastatic	I	34	Binimetinib	Reported median PFS of 1.9 months and median OS of 5.3 months	Single-arm dose-escalation study, documented dose-limiting toxicities at first dose-levels, requiring de-escalation	NCT04132505	[33]
Previously treated, metastatic	II	10	5-Fluorouracil Gemcitabine Devimistat [1]	Reported median PFS of 1.7 months and median OS of 3.1 months	Single-arm study, documented no additional safety signals from devimistat and HCQ	NCT05733000	[31]
Previously treated, metastatic	II	20	None	Reported median PFS of 46.5 days and median OS of 69.0 days	Single-arm study, documented serious adverse events in 10% of patients	NCT01273805	[27]
Previously treated, metastatic	II	36	LY3214996	Reported median PFS of 1.3 *vs*. 1.9 months and median OS of 2.4 *vs*. 4.6 months in the HCQ *vs*. non-HCQ arm	Two-arm study, documented serious adverse events in 30% *vs*. 25% in the HCQ *vs*. non-HCQ arm	NCT04386057	[29]
Previously untreated, metastatic	I	30	Gemcitabine Nab-paclitaxel Ipilimumab Nivolumab [1]	Reported 12-month OS rates of 53.9% *vs*. 65.5% in the HCQ *vs*. nivolumab arm	Two-arm study, documented serious adverse events in 53% of patients receiving HCQ	NCT04787991	[34]
Resectable	I/II	35	Gemcitabine	Reported CA19-9 normalization in 61% of patients and median OS of 34.8 months	Single-arm study, documented no dose-limiting toxicities as defined in the study protocol	NCT01128296	[21]
Resectable	II	32	Gemcitabine Nab-paclitaxel Avelumab [1]	Reported no significant differences in pathological response or CA19-9 reduction rates across study arms	Two-arm study, documented serious adverse events in >65% of patients	NCT03344172	N/A
Resectable	II	50	Radiotherapy Capecitabine Gemcitabine	Reported median PFS of 11.7 months and median OS of 23.3 months	Single-arm study, documented serious adverse events in 4% of patients	NCT01494155	[30]
Resectable	II	64	Gemcitabine nab-Paclitaxel	Reported no significant differences in PFS or OS across study arms, but improved histopathologic responses in the HCQ *vs*. non-HCQ arm	Two-arm study, reported no differences in serious adverse events across study arms	NCT01978184	[23]

Abbreviations: HCQ, hydroxychloroquine; mFOLFIRINOX, modified folinic acid, 5-fluorouracil, irinotecan and oxaliplatin; N/A, not available or not applicable; PDAC, pancreatic ductal adenocarcinoma; PFS, progression-free survival; OS, overall survival. [1] not in all study groups [2]; as of 6/20/26.

These observations highlight various persisting challenges that must be overcome to effectively move the concept of autophagy inhibition from a promising preclinical entity to a clinical reality for patients with PDAC (and other hard-to-treat tumor types). First, currently available pharmacological inhibitors of autophagy suffer from considerable specificity issues. As a standalone example, CQ, HCQ and their derivatives target lysosomal functions at large, *de facto* influencing a number of vesicular trafficking pathways other than autophagy, which at least in part may explain their suboptimal safety profile [1]. Notably, HCQ potently inhibits macropinocytosis [35], a nutrient scavenging pathway that supports the survival of PDAC cells in response to glutamine deprivation and therapeutic challenges [35–37], but at the same time is fundamental for the survival and function of multiple healthy cells [38]. Second, in at least some clinical trials, autophagy inhibition by tolerable HCQ doses appeared to be inconsistent [27], casting doubts on the actual existence of a clinically viable therapeutic window for this class of compounds. Third, even hitherto experimental drugs that more selectively target components of the autophagy machinery, *e.g*., ULK1 (unc-51 like autophagy activating kinase 1) inhibitors, are expected to influence other, non-autophagic ULK1-dependent processes, hence being prone to on-target unwarranted effects [39]. Fourth, human PDACs exhibit an extraordinary degree of intratumoral heterogeneity that is not appropriately recapitulated by currently available preclinical models [32]. This may account (at least partially) for the substantial discrepancy between the promising efficacy of autophagy inhibitors observed in preclinical PDAC models and the results of multiple clinical trials evaluating this strategy in patients with pancreatic cancer. Moreover, it suggests that future clinical studies should carefully consider the implementation of patient selection strategies based on the assessment of autophagy status in the TME.

Finally, autophagy is highly required for the survival and effector functions of multiple immune cells actively contributing to (natural and therapy-driven) cancer immunosurveillance [40], notably CD8^+^ cytotoxic T cells and NK (natural killer) cells [41]. Thus, nonselective autophagy inhibitors, even when delivered to the TME rather than systemically, may cause beneficial effects by targeting autophagy in malignant cells and the tumor stroma, but at the same time compromise therapeutically relevant anticancer immune responses [1]. This appears to be especially relevant for PDAC, as an abundant preclinical literature demonstrates that pancreatic CAFs (which rely on autophagy to support tumor growth via metabolic circuitries) [8,9,11], actively suppress NK cell-dependent immune responses [42,43]. While such a scenario stands out as particularly problematic for combining autophagy inhibitors with immunotherapeutics like immune checkpoint inhibitors, many conventional cancer treatments have also been shown to engage immune effector cells as part of their mechanism of action [40]. Thus, the detrimental effects of autophagy blockers on immune functions may also have contributed to the disappointing clinical findings summarized above.

Importantly, several new combinatorial regimens involving the inhibition of autophagy have recently been reported to mediate robust antineoplastic effects in preclinical PDAC models, including (but not limited to): (1) IGFR1 (insulin like growth factor 1 receptor) inhibitors plus ERK (extracellular signal-regulated kinase) inhibitors and HCQ [44], and (2) HSPA1A (heat shock protein family A (Hsp70) member 1A) inhibitors plus HCQ [45]. Whether these combinatorial paradigms may be safely and effectively translated to the clinic, however, remains to be determined. Along similar lines, whether simultaneously or sequentially targeting KRAS and autophagy in the context of immune checkpoint blockade, as supported by the notions that the efficacy of KRAS inhibitors involves an immunological component [46,47], and that blocking KRAS drives promotes a cytoprotective autophagic program [48], might successfully translate into a safe and effective strategy for patients with PDAC is unclear. Finally, whether mitophagy, a mitochondrion-specific variant of autophagy that has recently been shown to underlie resistance to KRAS inhibition and chemotherapy in preclinical PDAC models [10,49], can be pharmacologically targeted in a safe manner for therapeutic purposes remains to be formally demonstrated.

In conclusion, despite now more than two decades of intensive preclinical and clinical investigation, inhibiting autophagy for cancer therapy remains challenging [1], especially in indications as complex as pancreatic cancer. Indeed, while we surmise that overcoming the aforementioned obstacles will open new possibilities in this respect and potentially even unlock part of the therapeutic promise of this paradigm, autophagy sits at a very central position in the biology of all eukaryotic cells, hence representing a particularly difficult target for therapeutic exploitation.

## Data Availability

Data sharing is not applicable in this case as no new data has been created or analyzed in this study.
